# Association between total water intake and dietary intake of pregnant and breastfeeding women in China: a cross-sectional survey

**DOI:** 10.1186/s12884-019-2301-z

**Published:** 2019-05-15

**Authors:** Yalin Zhou, Xiaoyu Zhu, Yong Qin, Yong Li, Minjia Zhang, Wei Liu, Hanming Huang, Yajun Xu

**Affiliations:** 1 Department of Nutrition and Food Hygiene, School of Public Health, Peking University, NO.38 Xueyuan Road, Beijing, 100083 China; 2 Beijing Northern Hospital, NO.10 Chedaogou Road, Beijing, China; 3 Peking University Third Hospital, NO.49 Huayuan North Road, Beijing, 100191 China; 4 Beijing Key Laboratory of Toxicological Research and Risk Assessment for Food Safety, Peking University, NO.38 Xueyuan Road, Beijing, 100083 China

**Keywords:** Water intake, Pregnant, Breastfeeding, Hydration, Dietary intake, China

## Abstract

**Background:**

Pregnant and lactating women are at high risk of insufficient water intake. The cross-sectional study was mainly designed to evaluate the water intake, including total water intake (TWI), plain water intake, and water intake from beverages and foods of 200 pregnant women and 150 breastfeeding women in Beijing.

**Methods:**

A semi-quantitative Food Frequency Questionnaire (FFQ) was employed to assess their dietary intake, TWI, plain water, and water intake from beverages and foods. Multivariate regression analysis was conducted for evaluating the association between water intake and dietary variables.

**Results:**

On average, the TWI of pregnant and breastfeeding women was 2638 mL/day and 3218 mL/day, respectively. Only 28% of pregnant women and 27% of breastfeeding women were complied with the adequate intake (AI). Water from foods was the greatest contributor to TWI both in pregnant and breastfeeding women. TWI was positively related to some dietary variables (*P* < 0.001). For pregnant women, with each 100 kcal/day increase in energy intake, the TWI increased by 67 mL. With each 5 g increase in daily intake of dietary protein, fat, carbohydrate and fiber, TWI increased by 72 mL, 66 mL, 22 mL, 353 mL, respectively. When the energy contribution of protein increased by 5%, TWI increased by 210 mL. The each 100 mg increase in daily sodium intake was accompanied with 52 mL increase in TWI. For breastfeeding women, with each 100 kcal/day increase in energy intake, the TWI increased by 54 mL. With each 5 g increase in daily intake of dietary protein, fat, carbohydrate and fiber, TWI increased by 53 mL, 58 mL, 16 mL, 212 mL, respectively. The each 100 mg increase in daily sodium intake was accompanied with 54 mL increase in TWI.

**Conclusions:**

A large proportion of pregnant and breastfeeding women in Beijing were not adherent to AI for TWI set by Chinese Nutrition Society. Water intake from foods was the greatest contributor to TWI both in pregnant and breastfeeding women, and maternal dietary intake posed impacts on water intake during pregnancy and lactation. More researches are required to assess the water intake and hydration status of the populations.

**Electronic supplementary material:**

The online version of this article (10.1186/s12884-019-2301-z) contains supplementary material, which is available to authorized users.

## Background

Water plays an essential role in the bodily functions, modulating normal osmotic pressure, maintaining body temperature, and regulating biochemical metabolism [1]. It has been well recognized that water is one of critical factors closely associated with physical and mental functions, as well as chronic diseases [2, 3].

The balance between water intake and output is defined as hydration. Both excessive intake and insufficient intake exert a negative impact on body health. Compared with excessive intake, insufficient water intake—dehydration, is more common [4]. It has been reported that hydration of pregnant and breastfeeding women is linked with maternal or offspring health outcomes, which arouses awareness on TWI of women during pregnancy and lactation [5]. Due to special physical status, pregnant and breastfeeding women are always those who suffer a high risk of dehydration. During gestation, plasma volume experiences a physical expansion, and amniotic fluid from maternal body fluids maintains and protects the development of fetus. Pregnant women gain about 11 kg of body mass during the whole pregnancy, a large portion of which (approximately 7–8 L) comes from water retention [6]. It is documented that the maternal body water accretion is positively correlated with birth weight and the amount of amniotic fluid which makes a good prediction on the well-being of fetus. Investigators have found that dehydration of pregnant women contributes to adverse pregnancy outcomes, such as abortion, preterm, and preeclampsia [5, 7–10]. During the postpartum period, breastfeeding mothers experience an increased water loss via milk secretion, representing approximately 700 mL per day at 8 weeks postpartum [11, 12]. Although there is lack of tenable evidence to support the positive effect of increased water intake on milk production, substantial water loss via milk puts women at a high risk of dehydration, which lays an adverse effect on maternal health [11]. To promote appropriate water intake of pregnant and nursing women, many countries and institutions have set AIs for TWI of their own. The Institute of Medicine (IOM) established TWI at 3.0 L/day for pregnant women and 3.3 L/day for breastfeeding women [13]; European Food Safety Agency (EFSA) recommended 2.3 L/day for pregnant women and 2.7 L/day for breastfeeding women [14]; According to the recommendations of Chinese Nutrition Society, the AI was set at 3.0 L/day for pregnant women and 3.8 L/day for breastfeeding women [1].

The development of reference values for adequate TWI is based at least in part, if not entirely, on the sufficient survey data. The IOM set AI of TWI for pregnant and breastfeeding women on the basis of the National Health and Nutrition Examination Survey III (NHASE III). However, the EFSA had no European data available to observe water intake of pregnant and breastfeeding women. Given fact that energy intake increases by 300 kcal/day during gestation, it is suggested that pregnant women should increase 300 mL/day compared with those non-pregnant. Likewise, for breastfeeding women, AI of TWI recommended by the EFSA was also based on theoretical evidence that breastfeeding women produce milk of 700 ml/day, so water intake needs to increase 700 mL/day to make corresponding compensation for the loss via milk. To our best known, TWI of pregnant and breastfeeding women has not been well studied. Currently, only two studies on maternal total fluids intake (TFI) have been published. One was conducted in Indonesia, and the other was in Mexico [15–17]. However, both of the studies reported TFI (plain water and beverages) of pregnant and breastfeeding women without information of water intake from foods. It is well known that women tend to have extremely different dietary patterns during gestation and lactation, which inevitably influences water intake from foods and changes the contribution of water from different sources to TWI. In China, there is also lack of data available on TWI among pregnant and breastfeeding women.

Therefore, more surveys on TWI should be conducted to provide data for future revision of the AI of TWI for pregnant and breastfeeding women. The study is designed (1) to describe the TWI including plain water, water from beverages and foods; (2) to compare actual TWI with AI of TWI set by Chinese Nutrition Society; (3) to investigate water intake in relation to dietary characteristics.

## Methods

### Data source and study sample

The sample size was calculated by using the formula: N = (*Z*_α_*S)^2^/ *d*^2^. *Z* is the value associated with the desired confidence level; *S* is the estimated standard deviation in the population (based on the previous study); *d* is the maximum measurement error allowed by the researcher. For our calculation, the value of *S* was taken from the results reported in a previous study which showed a standard deviation of 472 mL of daily plain water intake for women (18-55y) [1]. In addition, the study was a part of a multinational study called Liq.In7 (Liquid Intake over 7 days). The value of *Z* was set for a confidence level of 95% (*Z*_α_ = 1.96), and the maximum allowable error set by ourselves (*d*) was 100 mL. The sample size should be 94 at least via the calculation. The cross-sectional survey was conducted in Beijing. Data collection was performed from February 2018 to July 2018. The study enrolled pregnant and breastfeeding women in the selected maternity hospital. Eventually, 200 pregnant women and 150 breastfeeding women eligible for inclusion criteria were recruited, which satisfies the demand of sample size.

This study was carried out according to the guidelines laid down in the Declaration of Helsinki and all procedures involving human subjects were approved by the Committee on Medical Ethics of the Peking University, with the number of ethics approval of IRB00001052–17107. Written informed consent was obtained from all subjects.

Inclusion criteria for pregnant women included having signed the informed consent, having age above 18 years, being primigravid, having no pregnancy complications such as hyperemesis gravidarum, hypertension or (gestational) diabetes based on interview and physical examination. Inclusion criteria for lactating women included being in the first semester of lactation, regardless of whether this was exclusive or complemented by formula feed, being apparently healthy with no acute or chronic diseases. The exclusion criteria were being illiterate or having difficulty with oral communication.

### Water intake assessment

The data on fluids intake was collected by trained interviewers via a FFQ. Standard containers were provided as visual aids to help participants recall water intake accurately. In accordance with the General Standard for the Beverages (GB/T10789–2015), the fluids were divided into the six categories: milk, botanical protein drinks, hot tea and coffee, alcoholic beverages, soft sweetened beverages (SSBs), 100% fruit juices. The SSBs were further classified into four subgroups: carbonated drinks, fruit drinks, milk drinks, and functional drinks [18].

In the present survey, four variables were used to assess the water intake: (1) plain water intake (including tap water and bottled water); (2) water from beverages; (3) water from foods; (4) TWI (including plain water, water from beverages and foods).

### Dietary intake

The semi-quantitative FFQ, with 48 food items included, was used for querying about diet of subjects. Women were instructed to complete the FFQ including frequency (per day/week/month) and food consumption. Food models and pictures were provided as visual aids to help participants recall food portion sizes accurately. The frequency of intake was transformed into daily level, and portion sizes into g or mL. The daily amounts of each food item were calculated by multiplying the daily frequency by the portion sizes. Daily nutrients and energy intake were analyzed with the nutrition analysis system (Shanghai Gongrong Medical Science and Technology co., LTD).

### Potential covariates

Potential covariates were adjusted in multiple liner regression models to evaluate independent association between water intake and dietary variables. Socio-demographic variables included age (continuous variables), body mass index (BMI, kg/m^2^, categorized variables: underweight, normal weight, overweight, obesity), working status (housewife, government employment, professionals, service, other), educational level (high school or below, college, postgraduate or up), physical activity (low, middle, high), gestational weeks for pregnant women (first trimester, second trimester, third trimester) and feeding modes for breastfeeding women (breastfeeding, mixed feeding).

### Statistical analysis

The demographic and anthropometric characteristics of the subjects were presented as mean and standard deviations for continuous variables, number and percentages for categorized variables. Due to the skewed distribution of data on water intake, water intake variables were both presented as mean and standard deviation, and median and percentile 25th and 75th. Comparison of water intake by demographic and anthropometric variables was evaluated with the Wilcoxon rank chi-square test.

Multiple linear regressions were conducted to explore independent association between water intake and dietary intake (intake of food categories and nutrients) after adjustment for potential covariates. As for the analysis, water intake included four water intake variables above mentioned.

All statistical tests were two-tailed and the significant level were set at *P* < 0.05. These analyses were performed by using the SPSS software version 22.0 (SPSS Inc., Chicago, IL, USA).

## Results

### Sample description

The survey was conducted among 350 women including 200 pregnant women and 150 lactating women. Data presented in Table 1 demonstrates baseline characteristics of the subjects. The pregnant women was 28.7 years old on average, and their average BMI was 21.4 kg/m^2^; 13% of them were underweight; over half (69%) of pregnant women had normal pre-pregnancy BMI; 18% of them were overweight or obesity. Most of them (61%) graduated from college and 30% were housewives. The proportion of gestational trimesters was 38% for the early gestation, 33% for the second trimester, and 29% for the late pregnancy. In the sample of breastfeeding women, the average maternal age was 31 years and average BMI was 23.0 kg/m^2^. 4% of them were underweight; the women with normal BMI accounted for the highest proportion at 61%; almost 35% of lactating women were overweight or obesity. Almost all of them (93%) had higher educational level of college or up, and 38% of lactating women got involved in government employment. 52% of them exclusively or predominately breastfed their baby and 48% of them selected mixed feeding. The general characteristics of breastfeeding women grouped by feeding modes were shown in Additional file 1: Table S1. We observed no statistical difference in age, BMI, BMI classification, working status, education level, and physical activity between two groups.

**Table 1:** General characteristics of the pregnant and breastfeeding women^a^

Variables	Pregnant women	Breastfeeding women
sample size	200	150
Age (years)	28.7 (4.0)	31.9 (4.1)
BMI (kg/m^2^)	21.4 (4.0)	23 (3.7)
BMI classification^b^		
Underweight	25 (13)	7 (4)
Normal	138 (69)	91 (61)
Overweight	21 (11)	39 (26)
Obesity	12 (7)	12 (9)
Working Status		
Housewife	59 (30)	12 (8)
Government employment	28 (14)	57 (38)
Professionals	53 (27)	55 (36)
Service	37 (19)	22 (15)
Other	18 (9)	4 (2)
Education Level		
High School or below	56 (28)	11 (7)
College	121 (61)	74 (49)
Postgraduate or up	23 (11)	65 (44)
Physical activity		
Low	52 (26)	82 (55)
Middle	100 (50)	24 (16)
High	48 (24)	44 (29)
Gestational week		
First trimester	76 (38)	–
Second trimester	65 (33)	–
Third trimester	59 (29)	–
Feeding modes		
Breastfeeding	–	79 (52)
Mixed feeding	–	71 (48)

^a^Continuous data were presented as mean (SD) and categorized variables (including BMI classification, working status, educational level, and physical activity and gestational trimesters) as n (%).

^b^BMI was calculated with pre-pregnancy weight for pregnant women, and current weight for breastfeeding women

### TWI and daily water intake from different sources

The mean and distribution percentiles of daily TWI and water intake from different sources are presented in Table 2. The mean TWI of pregnant and breastfeeding women was 2638 ± 1047 mL/day and 3218 ± 1254 mL/day, respectively. In the sample of pregnant women, water intake from foods stood at the highest intake of 1266 ± 711 mL. The water consumption from plain water and beverages was recorded as 1160 ± 535 mL and 225 ± 201 mL, respectively. Similarly, breastfeeding women had highest water intake from foods (1472 ± 709 mL), while the plain water intake and water intake from beverages were 1449 ± 967 mL and 298 ± 277 mL, respectively.

**Table 2:** Total water and water from different sources intake of the pregnant and breastfeeding women

Source of water	Consumers	Mean (SD)	Percentiles						
			5	10	25	50	75	90	95
Pregnant women									
Total Water Intake	200 (100)	2638 (1047)	1269	1475	2005	2539	3147	3929	4717
Plain Water	200 (100)	1160 (711)	300	500	800	1000	1500	2000	2600
Water from beverages	200 (100)	225 (201)	3	34	89	178	309	435	576
Water from foods	200 (100)	1266 (535)	490	609	853	1121	1413	1924	2284
Breastfeeding women									
Total Water Intake	150 (100)	3218 (1254)	1585	1897	2391	2901	3931	5007	5959
Plain Water	150 (100)	1449 (967)	300	500	800	1200	2000	2900	3000
Water from beverages	150 (100)	298 (277)	0	15	109	245	391	619	949
Water from food	150 (100)	1472 (709)	1585	1897	2391	2901	3931	5007	5959

Additional file 1: Table S2 and Table S3 in shows TWI and water from different sources intake of the pregnant and breastfeeding women categorized by BMI (pre-pregnancy BMI for pregnant women, current BMI for breastfeeding women), working status, educational level, physical activity, gestational weeks (for pregnant women), and feeding modes (for breastfeeding women). No significant difference was seen in TWI, plain water intake, and water intake from beverages among pregnant and breastfeeding women grouped by categorized variables as mentioned above, while water intake from foods showed significant difference among gestational trimesters in the sample of pregnant women (*P* = 0.041).

### Comparison with AI of TWI for pregnant and breastfeeding women set by Chinese nutrition society

**Fig. 1:**
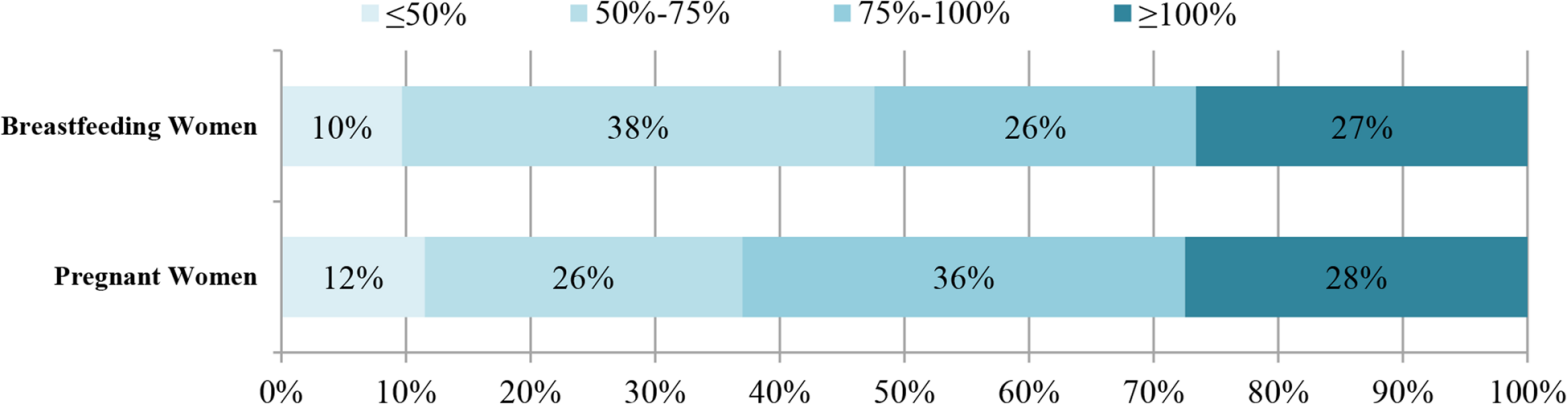
Total water intake (TWI) of pregnant and breastfeeding women. Figure 1 shows the proportion of subjects consuming ≥100, 75–100%, 50–75%, and ≤ 50% of AIs of TWI set by Chinese Nutrition Society

Figure 1 manifests the proportion of subjects consuming ≥100, 75–100%, 50–75%, and ≤ 50% of AI for TWI set by Chinese Nutrition Society. Among pregnant women, only 28% was complied with the AI, while other 72% had no adequate TWI. As for breastfeeding women, the proportion of women adherent to the AI was only 27%. Non-adherence to the AI of TWI was observed among 73% of the total breastfeeding women.

### The contribution of water from different sources to TWI

**Fig. 2:**
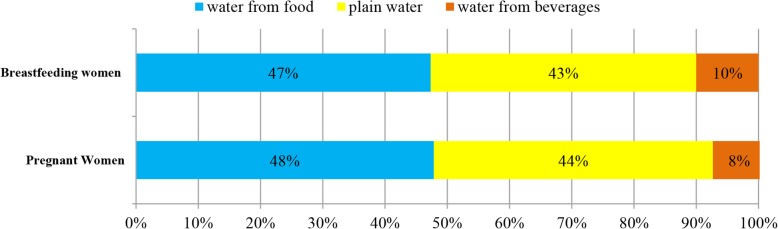
The contribution (%) of water intake from different sources to TWI. Figure 2 illustrates the contribution of water intake from different sources to TWI among pregnant and breastfeeding women

Figure 2 gives an illustration to the contribution of water intake from different sources to TWI among pregnant and breastfeeding women. The greatest contributor to TWI was water from foods whether in pregnant women (48%) or breastfeeding women (47%). Among pregnant women, the contribution of plain water and water from beverages to TWI was recorded as 44 and 8%, respectively. In the sample of breastfeeding women, plain water and water from beverages and foods accounted for 43 and 10%, respectively.

**Fig. 3:**
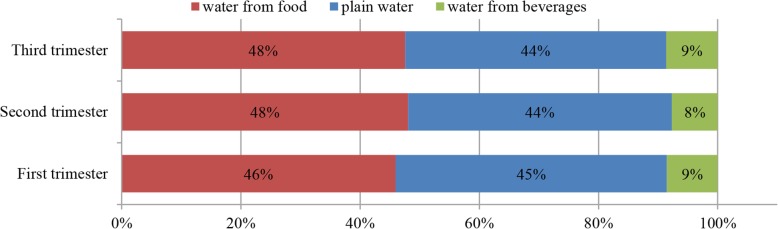
The contribution (%) of water intake from different sources to TWI among pregnant women. Figure 3 shows the contribution of water intake from different sources to TWI among pregnant women categorized by gestational trimesters

Figure 3 shows the contribution of water intake from different sources to TWI among pregnant women categorized by gestational trimesters. The chi-square test showed no significant difference in the contribution among women during different gestational trimesters.

**Fig. 4:**
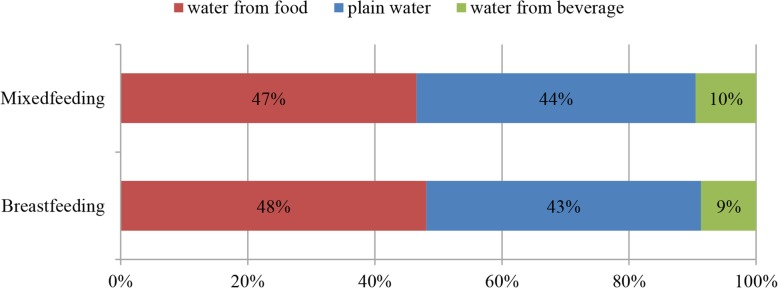
The contribution (%) of water intake from different sources to TWI among breastfeeding women. Figure 4 presents the contribution to water intake from different sources to TWI among breastfeeding women stratified by feeding modes

The contribution of water intake from different sources to TWI among breastfeeding women categorized by feeding modes is presented in Fig. 4. The contribution of water intake from different sources to TWI was similar among breastfeeding women stratified by feeding modes (shown in Fig. 4). The chi-square test showed no significant difference in the contribution among women in the groups of different feeding modes.

### The association between water intake from different sources and dietary characteristics among pregnant women

The crude association between water intake related variables and dietary characteristics are shown in Additional file 1: Table S4. We observed significant relationship between water intake variables and dietary variables with no covariates adjusted. After adjustment for potential covariates including age, BMI, working status, educational level, physical activity and gestational weeks, the independent association between water intake from different sources and dietary characteristics among pregnant women is presented in Table 3. Total daily energy intake showed significantly positive association with TWI, plain water, and water intake from beverages and food (*P* < 0.001); for each 100 kcal increment in energy intake, there is an increase of 67 mL, 21 mL, 12 mL, 37 mL in TWI, plain water intake, and water intake from beverages and foods, respectively. Daily protein intake, and fiber intake were positively correlated with all the water intake variables (*P* < 0.05), while fat intake only showed positive association with water intake from beverages (*P* < 0.001). TWI, water intake from beverages and foods increased with the rise in daily carbohydrate intake (*P* < 0.05). With increment in dietary sodium intake (*P* < 0.001), there was an uptrend in water intake variables except plain water intake. As for the energy from macronutrients, positive association between energy contribution of protein and TWI was observed (*P* = 0.044). Water intake from beverages showed positive association with energy contribution of fat, while water from foods was negatively correlated with that. When energy contribution of carbohydrate saw a 5% increase, there would be 40 mL increase in water intake from foods.

**Table 3:** The association between water intake from different sources and dietary characteristics among pregnant women

Variables	Total water		Plain water		Water from beverage		Water from food	
	β(95%CI)^d^	P^e^	β(95%CI)^d^	P^e^	β(95%CI)^d^	P^e^	β(95%CI)^d^	P^e^
Energy (100 kcal)^c^	67 (48,86)	< 0.001**	21 (6,36)	<0.001**	12 (8,16)	< 0.001**	37 (27,46)	< 0.001**
Protein(5 g)^cf^	72 (52,91)	< 0.001**	24 (9,39)	<0.001**	11 (7,15)	< 0.001**	39 (29,49)	< 0.001**
Fat(5 g)^cf^	66 (42,91)	< 0.001**	31 (13,48)	<0.001**	13 (9,18)	< 0.001**	23 (10,36)	< 0.001**
Carbohydrate(5 g)^cf^	22 (15,29)	< 0.001**	4(−1,10)	0.096	4 (2,5)	< 0.001**	15 (12,18)	< 0.001**
Fiber(5 g)^cf^	353 (283,423)	< 0.001**	62(1,124)	0.048*	38 (22,54)	< 0.001**	269 (247,291)	< 0.001**
Na^+^(100 mg)^cf^	52 (32,73)	< 0.001**	20 (5,35)	<0.001**	9 (5,13)	< 0.001**	24 (13,34)	< 0.001**
Energy from protein (5%)^c^	210(6,413)	0.044*	127(−14,268)	0.078	9(−30,49)	0.641	83(−22,188)	0.120
Energy from fat (5%)^c^	39(−63,141)	0.449	76(7,146)	0.031*	22 (3,42)	0.025*	−69(−120,-17)	< 0.001**
Energy from carbohydrate (5%)^c^	−27(−97,43)	0.449	− 51(− 99,-3)	0.038*	−12(−26,1)	0.077	40 (4,75)	0.030*

^c^The multiple liner regression models were adjusted for age (continuous), BMI (categorized variables), working status (housewife, government employment, professionals, service, other), educational level (high school or below, college, graduate or up), physical activity (low, middle, high), and gestational weeks (first, second and third trimester) for pregnant women;

^d^All values represented βs (95%CI) which were associated with units of measurement given in parentheses for each independent variable (for example, when there was a 100 kcal/day increase in energy intake, TWI would increase by 67 mL, plain water intake 21 mL, water intake from beverages 12 mL, and water intake from foods 37 mL);

^e^*P* Values obtained from the multiple linear regression analyses indicate the significance of the association of each independent variable with all water variables; ** *P *Values < 0.001; * *P* Values < 0.05

^f^besides the covariates as above mentioned, the models also included total daily energy (continuous) intake as an independent variable

### The association between water intake from different sources and dietary characteristics among breastfeeding women

As for breastfeeding women, the crude association between water intake related variables and dietary characteristics are shown in Additional file 1: Table S5. We observed significant relationship between water intake variables and dietary variables with no covariates adjusted. After adjustment for potential covariates including age, BMI, working status, educational level, physical activity and feeding modes, Table 4 shows the independent association between their water intake from different sources and dietary characteristics. Total daily energy intake showed significantly positive association with TWI, water intake from beverages and foods (*P* < 0.001); for each 100 kcal increment in energy intake, the 54 mL, 12 mL, 47 mL increased in TWI, and water intake from beverages and foods, respectively. Daily intake of protein, fat, carbohydrate and fiber intake were positively correlated with water intake variables (*P* < 0.001) except plain water. With increment in dietary sodium intake, there was an uptrend in water intake variables excluding plain water intake (*P* < 0.001). As for the energy from macronutrients, Water intake from beverages showed positive association with energy contribution of fat (*P* < 0.001). When the energy contribution of fat increased by 5%, there was would be 38 mL increase in water intake from beverages.

**Table 4:** The association between water intake from different sources and dietary characteristics among breastfeeding women ^g^

Variables	Total water		Plain water		Water from beverage		Water from food	
	β(95%CI)^h^	P^i^	β(95%CI)^h^	P^i^	β(95%CI)^h^	P^i^	β(95%CI)^h^	P^i^
Energy (100 kcal)^g^	54 (34,75)	< 0.001**	5(− 22,13)	0.612	12 (8,16)	< 0.001**	47 (37,57)	< 0.001**
Protein(5 g)^gj^	53 (34,72)	< 0.001**	2(−14,19)	0.470	10 (5,14)	< 0.001**	41 (32,51)	< 0.001**
Fat(5 g)^gj^	58 (30,87)	< 0.001**	−5(−28,19)	0.953	17 (11,23)	< 0.001**	46 (31,61)	< 0.001**
Carbohydrate(5 g)^gj^	16 (9,23)	< 0.001**	−2(−8,4)	0.213	3 (2,4)	< 0.001**	15 (12,18)	< 0.001**
Fiber(5 g)^gj^	212 (142,281)	< 0.001**	−2(−63,60)	0.766	24 (8,40)	< 0.001**	190 (161,219)	< 0.001**
Na^+^(100 mg)^gj^	54 (26,83)	< 0.001**	−15(−38,8)	0.693	15 (10,21)	< 0.001**	53 (39,67)	< 0.001**
Energy from protein (5%)^g^	208(− 136,553)	0.234	173(−92,439)	0.197	−25(−98,47)	0.487	41 (5)	0.547
Energy from fat (5%)^g^	−35(− 208,137)	0.686	−8(− 140,125)	0.910	38 (3,74)	0.034*	46 (8)	0.182
Energy from carbohydrate (5%)^g^	15(−104,134)	0.808	−10(− 102,82)	0.831	−17(−42,7)	0.165	15 (2)	0.220

^g^The multiple liner regression models were adjusted for age (continuous), BMI (categorized variables), working status (housewife, government employment, professionals, service, other), educational level (high school or below, college, graduate or up), physical activity (low, middle, high), and feeding modes (mixed feeding, breastfeeding); ^*h*^ All values represented βs (95%CI) which were associated with units of measurement given in parentheses for each independent variable (for example, when there was a 100 kcal/day increase in energy intake, TWI would increase by 54 mL, plain water intake 5 mL, water intake from beverages 12 mL, and water intake from foods 47 mL); ^*i*^ *P* Values obtained from the multiple linear regression analyses indicated the significance of the association of each independent variable with all water variables, ** *P* Values < 0.001; *P Values < 0.05; ^j^ besides the covariates as above mentioned, total daily energy (continuous) intake was also included in the model as an independent variable

## Disscusion

In the survey, pregnant and breastfeeding women in Beijing were recruited to obtain the data on water intake, evaluate maternal adherence to AI for TWI, and explore the independent association between water intake variables and dietary characteristics. The data acquired would sereve as one of reference baseline data for the further revision of AI of TWI among the population in China.

Compared with general population, pregnant and breastfeeding women tend to suffer a higher risk of dehydration because of maternal special physical status and mounting demands [9]. It is well documented that maternal dehydration poses a negative impact on maternal and offspring health [8, 19, 20]. Therefore, the study on the water intake of pregnant and breastfeeding women should be taken into consideration. Althoug AI for TWI among them has been set by countries and institutes in the world, there is still lack of data on actual water intaker in pregnant and breastfeeding women, which inhibits the further revision of these AIs. To our knowledge, this is the first survey on the water intake (including TWI, plain water, and water intake from beverages and foods) of Chinese pregnant and breastfeeding women. In our study, pregnant women consumed 2.6 L/day of total water (including 1.1 L of plain water, 0.2 L of water from beverages, and 1.3 L of water from foods) on average. As for breastfeeding women, the daily consumption of total water was 3.2 L/day (including 1.4 L of plain water, 0.3 L of water from beverages, and 1.5 L of water from foods). Three studies have been conducted among the same target population in Indonesia, Mexico, and Greek. However, all of previous surveys only foucused on the TFI, without the water from foods taken into account. Therefore, our findings cannot be comparable with the previous results. Nevertheless, the recommended AI of TWI provides us with a reference. Comparing with reference values (2.3 L for pregant women and 2.7 L for breastfeeding women) set by the EFSA, we found the TWI of pregnant and breastfeeding women were desirable. Referring to recommended values from the IOM, it was turned out that breastfeeding women showed good adherence rather than pregnant women. Based on the standard of 2.7 L of AI for general female adults set by Chinese Nutrition Society, Chinese pregnant women are recommened to increase TWI by 0.3 L/day, and lactating women should consume more 1.1 L/ day for meeting the demands of mothers and children. Regrettably, in the current study, the TWI of pregnant women cannot satisfy the needs of general women population. What’s more, the average TWI of breastfeeeding women is higher than that of general female adults rather than complying with AI of lactating women. Among pregnant and breastfeeding women in Beijing, less than a half of the targeted population (28% for pregnant women, and 27% for breastfeeding women) were adherent to the AI for TWI desgined by Chinese Nutrition Society. Therefore, it is indicated that these pregnant and breastfeeding women might be at a risk of being under-hydrated.

Assessing the contribution of water intake from different sources to TWI, we observed that the greatest contributor of TWI was water intake from foods, followed by plain water, which was extremely different from results in previous studies performed in some western countries. In some previous studies, daily plain water intake accounted for about 80% of TWI, while water intake from foods only contributed to 20% [3, 14, 21]. For example, Athanasatou.et al. found the plain water and water intake from foods to TWI accounted for about 78 and 22%, respectively, in Greek adults [22]. EFSA reported the contribution of plain water to TWI was 70%~ 80%, and that of water intake from foods only 20%~ 30% in England and India [23]. However, Ma.et al. reported that 40% of daily total water consumed by Chinese adults came from food, which gave a support to our findings [24]. The discrepancy implied that geographical location, climate and diet culture might be a matter of great account. It is well known that the major Chinese cooking styles, that is, steaming and stewing, make to maintain the most moisture in foods [25]. On the other hand, plant foods recognized as good sources of water are dominant in the Chinese traditional dietary patterns. Actually, slight difference from that of Ma was shown in the study, which informed us of variance of water intake from different sources among diverse population. Ma et al. recruited general population as the subjects, while we focused on the specific population—pregnant and breastfeeding women. Pregnant and lactating women are recommended to increase dietary and nutrients intake properly by Chines Nutrition Society so as to meet maternal and offspring demands. In addition, it is generally believed that more consumption of soup will be beneficial to milk production in accordance with Chinese traditional notions. These might explain the reason why water intake from foods made up for the largest proportion of TWI.

In the process of exploring the relationship between water intake and dietary characteristics, we observed daily dietary energy intake showed a positive association with TWI, water intake from beverages and foods, which was consistent with the findings in another survey on correlation between water intake and dietary variables among Korean adults [18]. In our study, higher consumption of protein was associated with increment in TWI and water intake from foods. The higher TWI came along with the higher energy contribution from protein among pregnant women in the study. Sui et al. reported that TWI increased as energy contribution of protein rose among Australian population [2]. Similarly, Lee and his colleagues also found the positive correlation among Korean adults. In animals’ experiments, high intake of dietary protein resulted in the increase of water intake and the volume of urine. Urea is the terminal products of protein metabolism, and 40~60 mL water needed for 2.2 g urea. In addition, high protein intake would promote water intake by increasing osmotic pressure of plasma [3]. A better diet quality is beneficial to healthy drinking patterns. Higher consumption of dietary fiber, fruits and vegetables have a positive impact on TWI, plain water and water intake from foods. According to our survey, high-fat diets increased the water intake from beverages. The eating behaviors might be one of reasons. It was reported that people tended to consume more beverages, soft sweetened beverages in particular, while eating fast food or snacks which are known as high-fat-content foods. As an important mineral element theoretically influencing water intake, dietary sodium has always been the focus. High intake of sodium promotes water intake by stimulating thirst to maintain water homeostasis in our body [26]. The finding of our survey was that high intake of dietary sodium of pregnant women had a positive association with all components of water intake. However, breastfeeding women who consumed more dietary sodium had higher intake of TWI, water intake from beverages and foods except plain water. Kant et al. reported that dietary sodium intake was positively associated with water from foods, rather than water from beverages among American adolescents [3]. Lee et al. also reported that dietary sodium was positively correlated with all components of water intake (TWI, plain water intake, and water intake from beverages and foods). Different cultural background might result in the divergent results [23].

We acknowledged that there were some limitations. First, the survey was performed in Beijing, the capital of China. There are great differences in factors concerning water intake, including ambient temperature, climate, and cultural traditions among diverse regions in China. Therefore, our findings only represented the water intake of pregnant women and breastfeeding women in Beijing. What’s more, the participants were recruited in the particular maternity hospital, which led to a lack of random selection. As a result, the extrapolation of conclusion was limited. Second, this was a cross-sectional rather than a longitudinal survey, so we could not follow the subjects from pre-pregnancy throughout pregnancy into postpartum. Comparison of water intake between pregnancy trimesters should be cautious. On the other hand, we could not obtain the causal relationship between water intake and dietary variables from the cross-sectional survey. More and profound researches are required. Finally, dietary and water intake information and some potential covariates such as physical activity, pre-pregnant weight for pregnant women were recalled by participants, which would inevitably generate recall bias. Another limitation that should not be ignored was that we simplified the items concerning physical activities on the basis of the standard questionnaire (international physical activity questionnaire, IPAQ) rather than using the standard questionnaire to make an analysis on the physical activities of respondents.

In spite of limitations discussed above, the current survey had some merits of its own. To our best known, this is the first survey on the water intake among specific target populations, which provides baseline data on actual water intake of pregnant and breastfeeding women. Furthermore, a relatively large sample of pregnant women was enrolled, with equal distribution over pregnancy trimesters. As for breastfeeding, we recruited women during the first semester of lactation during which breastfeeding and mixed feeding are two major feeding styles. Therefore, some uncontrollable cofounders related to feeding modes can be avoided with no influence on our results. Last but absolutely not the least, we provided a photographic booklets and standard containers in order to reduce recall bias.

## Conclusions

In conclusion, 28% of pregnant women and 27% of breastfeeding women in Beijing were adherent to AI of TWI set by Chinese Nutrition Society. Water intake from foods accounted for the largest proportion of TWI both in pregnant and breastfeeding women. Maternal water intake during pregnancy and lactation was influenced by dietary factors. Given the importance of adequate hydration status during pregnancy and lactation, our survey could contribute to baseline actual data for future revision of AI of TWI among the special populations.

## Additional file

Additional file 1: **Table S1.** General characteristics of breastfeeding women grouped by feeding modes. **Table S2.** Total water and water from different sources intake of the pregnant women categorized by body mass index, working status, educational level, physical level, and gestational weeks^d^. **Table S3.** Total water and water from different sources intake of the breastfeedingwomen categorized by body mass index, working status, educational level, physical level, and Feeding modes^f^. **Table S4.** The association between water intake from different sources and dietary characteristics among pregnant womenwithout adjustment for covariates. **Table S5.** The association between water intake from different sources and dietary characteristics among breastfeeding women without adjustment for covariates. (PDF 541 kb)

## Abbreviations

AI: Adequate intake
BMI: Body mass index
EFSA: European Food Safety Agency
FFQ: Food frequency questionnaire
IOM: Institute of Medicine
IPAQ: International physical activity questionnaire
SSBs: Soft sweetened beverages
TFI: Total fluids intake
TWI: Total water intake
