# Supplementary material for: Association between total water intake and dietary intake of pregnant and breastfeeding women in China: a cross-sectional survey
Source: BMC Pregnancy Childbirth. 2019 May 15;19:172. doi: 10.1186/s12884-019-2301-z (PMC6521377; doi:10.1186/s12884-019-2301-z)
Supplement: Supplementary file 1 — Table S1. General characteristics of breastfeeding women grouped by feeding modes. Table S2. Total water and water from different sources intake of the pregnant women categorized by body mass index, working status, educational level, physical level, and gestational weeksd. Table S3. Total water and water from different sources intake of the breastfeedingwomen categorized by body mass index, working status, educational level, physical level, and Feeding modesf. Table S4. The association between water intake from different sources and dietary characteristics among pregnant womenwithout adjustment for covariates. Table S5. The association between water intake from different sources and dietary characteristics among breastfeeding women without adjustment for covariates. (PDF 541 kb) [file 12884_2019_2301_MOESM1_ESM.pdf]

Table S1. General characteristics of breastfeeding women grouped by feeding modes [a]

Variables	Breastfeeding	Mixed feeding	P [c]
sample size	79	71	
Age (years)	31.4(3.7)	32.5(4.5)	0.315
BMI (kg/m^2) [b]	22.7(3.9)	23.4(3.43)	0.431
**BMI classification**			0.333
Underweight	7(9)	0(0)	
Normal	48(61)	44(62)	
Overweight	19(24)	20(28)	
Obesity	5(6)	7(10)	
**Working Status**			0.358
Housewife	5(6)	7(10)	
Government employment	28(35)	28(40)	
Professionals	32(41)	24(33)	
Service	14(18)	7(10)	
Other	0(0）	4(7)	
**Education Level**			0.158
High School or below	6(8)	5(7)	
College	32(41)	43(61)	
Postgraduate or up	41(52)	23(32)	
**Physical level**			0.494
Low	38(48)	44(62)	
Middle	15(19)	10(14)	
High	26(33)	17(24)	

[a] Continuous data were presented as mean (SD) and categorized variables (including BMI classification, working status, educational level, and physical level) as n (%). [b] BMI was calculated with current weight and height. [c] *P* was obtained from the independent sample T test for continuous variables (age), and chi-square test for categorized variables (BMI classification, working status, educational level, and physical level).

Table S2. Total water and water from different sources intake of the pregnant women categorized by body mass index, working status, educational level, physical level, and gestational weeks [d]

Group	Total	Total Water Intake		Plain Water		Water from beverages		Water from food	
		Median(P25-P75)	*P* [e]	Median(P25-P75)	*P* [e]	Median(P25-P75)	*P* [e]	Median(P25-P75)	*P* [e]
**BMI classification**			0.459		0.276		0.665		0.829
Underweight	25(13)	2621(2027-3126)		1200(750-1500)		193(90-355)		1264(836-1444)	
Normal	138(69)	2539(1993-3156)		1000(800-1500)		178(98-303)		1135(884-1492)	
Overweight	21(11)	2653(2137-3635)		1500(1000-1800)		176(83-411)		1132(895-1349)	
Obesity	12(6)	2481(1478-3465)		1300(800-2000)		145(54-370)		904(535-1311)	
**Working Status**			0.452		0.848		0.300		0.347
Housewife	59(30)	2539(2035-3002)		1200(600-1500)		179(84-294)		1140(812-1412)	
Government employment	28(14)	2493(1994-3422)		1000(925-1575)		143(75-284)		1041(743-1358)	
Professionals	53(27)	2681(2055-3322)		1000(860-1500)		204(124-329)		1221(884-1559)	
Service	37(19)	2395(1827-2985)		1000(650-1500)		176(99-345)		1059(902-1379)	
Other	18(9)	2707(1801-3389)		1000(650-1850)		124(62-329)		1231(916-1491)	
**Education Level**			0.236		0.364		0.886		0.373
High School or below	56(28)	2500(1780-3002)		1000(550-1500)		159(90-283)		1065(800-1343)	
College	121(61)	2611(2005-3252)		1000(800-1500)		186(96-314)		1140(915-1455)	
Postgraduate or up	23(11)	2632(2046-3926)		1100(1000-1500)		133(60-367)		1220(901-1819)	
**Physical level**			0.111		0.098		0.894		0.875
Low	52(26)	2736(2310-3501)		1200(1000-1550)		179(79-357)		1111(908-1447)	
Middle	100(50)	2460(1967-3071)		1000(650-1500)		178(115-306)		1158(868-1393)	
High	48(24)	2656(1831-3078)		1000(800-1750)		158(84-288)		1077(770-1408)	
**Gestational week**			0.282		0.527		0.375		0.041
First trimester	106(53)	2539(1827-3036)		1000(800-1500)		168(80-303)		1086(765-1367)	
Second trimester	65(33)	2632(2040-3147)		1200(800-1600)		179(88-309)		1172(971-1413)	
Third trimester	29(14)	2544(2181-3858)		1200(1000-1500)		217(123-367)		1232(901-1780)	

[d] The categorized variables represented as number and percentage; all values of water intake variables represented median and percentile 25th and 75th; [e] P Value obtained from the Kruskal-Wallis H (K) test indicated the significant difference between different groups.

Table S3. Total water and water from different sources intake of the breastfeeding women categorized by body mass index, working status, educational level, physical level, and Feeding modes [f]

Group	Total	Total Water Intake		Plain Water		Water from beverages		Water from food	
		Median(P25-P75)	*P* [g]	Median(P25-P75)	*P* [g]	Median(P25-P75)	*P* [g]	Median(P25-P75)	*P* [g]
**BMI classification**			0.976		0.524		0.464		0.771
Underweight	7(5)	3426(2283-4349)		1750(950-2250)		206(131-541)		1294(848-1730)	
Normal	91(61)	2889(2446-3592)		1200(800-1800)		225(69-360)		1308(1001-1800)	
Overweight	39(26)	2963(2164-4494)		1375(725-2000)		264(133-405)		1326(1027-1867)	
Obesity	12(8)	3021(2272-4363)		1000(470-2000)		368(187-518)		1390(1137-2157)	
**Working Status**			0.493		0.557		0.600		0.202
Housewife	12(8)	2806(2162-3785)		1500(300-2000)		200(42-367)		1278(1057-1566)	
Government employment	61(40)	3043(2544-4401)		1500(800-2000)		256(116-384)		1470(1108-1885)	
Professionals	55(36)	2889(2207-3679)		1000(800-1800)		303(116-476)		1216(945-1848)	
Service	22(15)	2880(2356-3774)		1063(775-2000)		177(41-384)		1268(906-1760)	
**Education Level**			0.491		0.347		0.199		0.739
High School or below	11(7)	2765(1804-3082)		1000(400-1500)		42(36-244)		1522(1188-1774)	
College	74(49)	2930(2312-4053)		1500(750-2000)		239(120-431)		1281(982-1802)	
Postgraduate or up	65(44)	3004(2501-4119)		1200(900-2000)		282(114-402)		1405(1026-1872)	
**Physical level**			0.851		0.682		0.171		0.743
Low	82(55)	2812(2273-4329)		1200(800-2000)		209(69-367)		1305(1112-1809)	
Middle	24(16)	2967(2547-3700)		1500(850-1800)		275(174-511)		1196(943-1813)	
High	44(29)	2920(2294-3345)		1000(800-1875)		294(118-454)		1328(992-1885)	
**Feeding modes**			0.297		0.345		0.406		0.831
Breastfeeding	79(52)	2809(2313-3610)		1000(800-1900)		225(73-356)		1296(1026-1821)	
Mixed feeding	71(48)	3037(2431-4148)		1500(800-2000)		277(117-438)		1312(1015-1848)	

[f] The categorized variables represented as number and percentage; all values of water intake variables represented median and percentile 25th and 75th; [g] P Value obtained from the Kruskal-Wallis H (K) test indicated the significant difference between different groups.

Table S4. The association between water intake from different sources and dietary characteristics among pregnant women without adjustment for covariates

	Total water		Plain water		Water from beverages		Water from food	
	β(95%CI) [i]	*P* [j]	*β(95%CI)* [i]	*P* [j]	*β(95%CI)* [i]	*P* [j]	*β(95%CI)* [i]	*P* [j]
Energy (100kcal) [h]	67(48,86)	**<0.01**	22(7,36)	**<0.01**	12(8,15)	**<0.01**	36(27,46)	**<0.01**
Protein(5g) [h]	74(55,92)	**<0.01**	26(11,40)	**<0.01**	11(7,14)	**<0.01**	40(31,49)	**<0.01**
Fat(5g) [h]	65(42,89)	**<0.01**	30(13,47)	**<0.01**	13(9,18)	**<0.01**	23(10,35)	**<0.01**
Carbohydrate(5g) [h]	22(15,28)	**<0.01**	5(0,10)	0.058	4(2,5)	**<0.01**	15(12,18)	**<0.01**
Fiber(5g) [h]	356(287,425)	**<0.01**	65(6,125)	**0.031**	35(19,50)	**<0.01**	271(249,292)	**<0.01**
Na^+(100mg) [h]	51(31,71)	**<0.01**	1(6,35)	**<0.01**	9(5,13)	**<0.01**	22(12,33)	**<0.01**
Energy from protein(5%) [h]	93(439,0)	**<0.01**	135(8,263)	**0.037**	5(-32,41)	0.804	116(21,211)	**0.016**
Energy from fat(5%) [h]	39(-60,138)	0.441	71(3,138)	**0.040**	22(3,41)	**0.022**	-63(-113,-13)	**0.014**
Energy from carbohydrate(5%) [h]	-37(-105,32)	0.292	-52(-98,-6)	**0.028**	-12(-25,1)	0.074	31(-3,66)	0.077
Energy (100kcal) [h]	67(48,86)	**<0.01**	22(7,36)	**<0.01**	12(8,15)	**<0.01**	36(27,46)	**<0.01**

[h] The simple liner regression models were established with water intake from different sources as dependent variables and dietary characteristics as independent variables. [i] All values represented βs (95%CI) which were associated with units of measurement given in parentheses for each independent variable (for example, when there was a 100 kcal/day increase in energy intake, TWI would increase by 67mL, plain water intake 22 mL, water intake from beverages 12mL, and water intake from foods 36mL); [j] P Values obtained from the simple linear regression analyses indicated the significance of the association of each independent variable with all water variables.

Table S5.The association between water intake from different sources and dietary characteristics among breastfeeding women without adjustment for covariates

	Total water		Plain water		Water from beverages		Water from food	
	β(95%CI) [l]	*P* [m]	*β(95%CI)* [l]	*P* [m]	*β(95%CI)* [l]	*P* [m]	*β(95%CI)* [l]	*P* [m]
Energy (100kcal) [k]	55(35,75)	**<0.01**	-4(-21,13)	0.642	12(8,17)	**<0.01**	46(37,56)	**<0.01**
Protein(5g) [k]	54(35,72)	**<0.01**	2(-14,18)	0.766	10(6,14)	**<0.01**	41(32,50)	**<0.01**
Fat(5g) [k]	58(31,85)	**<0.01**	-4(-27,18)	0.717	18(12,23)	**<0.01**	44(30,59)	**<0.01**
Carbohydrate(5g) [k]	16(10,23)	**<0.01**	-2(-7,4)	0.502	3(2,5)	**<0.01**	15(12,18)	**<0.01**
Fiber(5g) [k]	209(141,277)	**<0.01**	-5(-65,55)	0.872	24(8,41)	**<0.01**	189(162,217)	**<0.01**
Na^+(100mg) [k]	54(27,81)	**<0.01**	-1(-36,7)	0.191	16(11,22)	**<0.01**	52(39,65)	**<0.01**
Energy from protein (5%) [k]	199(-139,537)	0.247	159(-102,419)	0.230	-25(-100,50)	0.514	64(-127,256)	0.508
Energy from fat (5%) [k]	-17(-181,146)	0.833	-9(-135,117)	0.885	43(8,79)	**0.016**	-52(-144,40)	0.269
Energy from carbohydrate (5%) [k]	7(-107,122)	0.900	-7(-95,82)	0.879	-21(-46,4)	0.103	35(-30,99)	0.287

[k] The simple liner regression models were established with water intake from different sources as dependent variables and dietary characteristics as independent variables. [l] All values represented βs (95%CI) which were associated with units of measurement given in parentheses for each independent variable (for example, when there was a 100 kcal/day increase in energy intake, TWI would increase by 55mL, water intake from beverages 12mL, and water intake from foods 46mL); [m] P Values obtained from the simple linear regression analyses indicated the significance of the association of each independent variable with all water variables.
